# Role of PheE15 Gate in Ligand Entry and Nitric Oxide Detoxification Function of *Mycobacterium tuberculosis* Truncated Hemoglobin N

**DOI:** 10.1371/journal.pone.0049291

**Published:** 2012-11-08

**Authors:** Ana Oliveira, Sandeep Singh, Axel Bidon-Chanal, Flavio Forti, Marcelo A. Martí, Leonardo Boechi, Dario A. Estrin, Kanak L. Dikshit, F. Javier Luque

**Affiliations:** 1 Department of Physical Chemistry and Institute of Biomedicine (IBUB), Faculty of Pharmacy, University of Barcelona - Recinte Torribera, Santa Coloma de Gramenet, Spain; 2 CSIR-Institute of Microbial Technology, Chandigarh, India; 3 Departamento de Química Inorgánica, Analítica y Química Física/Instituto de Química Física de los Materiales, Medio Ambiente y Energía (INQUIMAE), Facultad de Ciencias Exactas y Naturales, Universidad de Buenos Aires, Buenos Aires, Argentina; German Research School for Simulation Science, Germany

## Abstract

The truncated hemoglobin N, HbN, of *Mycobacterium tuberculosis* is endowed with a potent nitric oxide dioxygenase (NOD) activity that allows it to relieve nitrosative stress and enhance *in vivo* survival of its host. Despite its small size, the protein matrix of HbN hosts a two-branched tunnel, consisting of orthogonal short and long channels, that connects the heme active site to the protein surface. A novel dual-path mechanism has been suggested to drive migration of O_2_ and NO to the distal heme cavity. While oxygen migrates mainly by the short path, a ligand-induced conformational change regulates opening of the long tunnel branch for NO, via a phenylalanine (PheE15) residue that acts as a gate. Site-directed mutagenesis and molecular simulations have been used to examine the gating role played by PheE15 in modulating the NOD function of HbN. Mutants carrying replacement of PheE15 with alanine, isoleucine, tyrosine and tryptophan have similar O_2_/CO association kinetics, but display significant reduction in their NOD function. Molecular simulations substantiated that mutation at the PheE15 gate confers significant changes in the long tunnel, and therefore may affect the migration of ligands. These results support the pivotal role of PheE15 gate in modulating the diffusion of NO via the long tunnel branch in the oxygenated protein, and hence the NOD function of HbN.

## Introduction

*Mycobacterium tuberculosis* (*Mtb*) poses a serious threat to the public health worldwide, infecting nearly one third of the global population. The remarkable adaptability of tubercle bacillus to cope with hazardous level of reactive nitrogen/oxygen species within the intracellular environment contributes to its pathogenicity. An enhanced level of nitric oxide (NO) and reactive nitrogen species produced within activated macrophages during infection act as a vital part of host defense, limit the intracellular survival of *Mtb*, and contributes in restricting the bacteria to latency. Nevertheless, *Mtb* has evolved efficient resistance mechanisms by which toxic effects of NO and nitrosative stress can be evaded. One of the unique defense mechanisms by which *Mtb* protects itself from the toxicity of NO relies on the oxygenated form of truncated hemoglobin N (HbN), which catalyzes the rapid oxidation of NO to harmless nitrate [1]–[3]. Compared to horse heart myoglobin, the nitric oxide dioxygenase (NOD) reaction catalyzed by *Mtb* HbN is ∼15-fold faster, suggesting that it may be crucial in relieving nitrosative stress [4].

**Figure 1:**
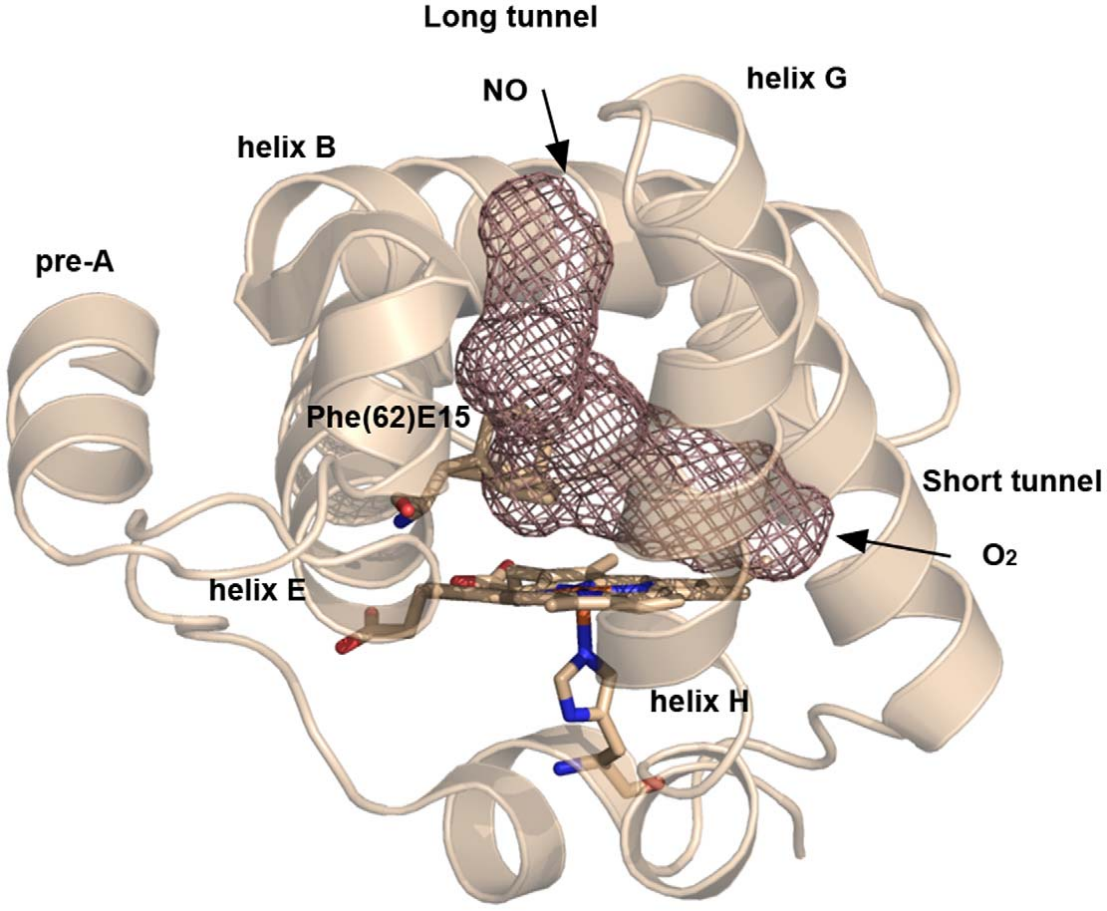
Representation of the long and short branches of the tunnel system. The graphical display is based on the X-ray crystallographic structure of *Mtb* HbN (PDB entry 1IDR), and the access routes of O_2_ and NO in the dual-path ligand-modulated mechanism proposed for this protein are indicated. The gating residue PheE15 (residue 62) is shown in the two conformations found in the X-ray structure as sticks.

Despite having single domain architecture, the NO-scavenging ability of *Mtb* HbN is comparable to flavoHbs that are integrated with a reductase domain and known to have a high NOD activity. It is thus important to understand what structural and dynamical features contribute to the efficiency of its enhanced NO-scavenging function, and therefore ensure survival of the bacillus under nitrosative stress. X-ray crystallographic studies revealed that *Mtb* HbN hosts a protein matrix tunnel composed by two orthogonal branches [5], [6]. In addition, computational simulations performed by some of the authors suggested that *Mtb* HbN has evolved a novel dual-path mechanism to drive migration of O_2_ and NO to the distal heme cavity [7], [8]. According to such a mechanism (Fig. 1), access of O_2_ to the heme cavity primarily involves migration through the tunnel short branch (∼10 Å long, shaped by residues in helices G and H). Binding to the heme then regulates opening of the tunnel long branch (∼20 Å long, mainly defined by helices B and E) through a ligand-induced conformational change of PheE15 residue, which would act as a gate. It has been recently shown that the opening of PheE15 in the oxygenated protein is also affected by the N-terminal Pre-A motif [9].

Since the NOD function of HbN depends on the diffusion of NO to the O_2_-bound heme through the long tunnel branch, the PheE15 gate emerges as a fundamental residue in determining the overall efficiency of the NO scavenging. Accordingly, the NOD function of HbN must result from a balanced tuning of the opening/closing events of the gate. Moreover, the functional implication of PheE15 in assisting the NOD activity is supported by the preservation of this residue in mycobacterial HbNs, while it is replaced by other residues in truncated hemoglobins O and P [10], [11]. However, to the best of our knowledge, no experimental data have yet been reported to examine the gating role of PheE15 and its influence on the NOD activity conducted by *Mtb* HbN. In this context, this study has been undertaken to probe the role of PheE15 in protein function. To this end, several PheE15 gate mutants have been tested experimentally for their NOD function. In addition, molecular dynamics (MD) simulations have been performed to analyze the structural changes in the topology of long tunnel and the alterations in the protein dynamics, paying attention to the ligand migration properties through the tunnel. Our results confirm the critical role played by E15 in ligand migration along the long channel.

## Materials and Methods

### Strains, Plasmids and Culture Conditions

*Escherichia coli* strains, JM109 and BL21DE3 were used for the cloning and expression of recombinant genes. Bacterial cultures were grown in Luria-Bertani (LB) or Terrific Broth (containing 24 g of Yeast Extract, 12 g of Bacto-Tryptone, 12.54 g of K_2_HPO_4_, 2.31 g of KH_2_PO_4_) medium at 37°C at 180 r.p.m. When required, ampicillin and kanamycin (Sigma) were added at a concentration of 100 and 30 µg/ml, respectively. Plasmids, pBluescript (Stratagene) and pET28C (Novagen) were used for cloning and expression of recombinant genes as described earlier [3], [12]. The oligonucleotides were custom synthesized by Integrated DNA Technologies Inc. NO (98.5%) was obtained from Sigma Aldrich and saturated NO was prepared as mentioned previously [13]. Heme content of the cell was measured as noted in previous studies [13].

**Figure 2:**
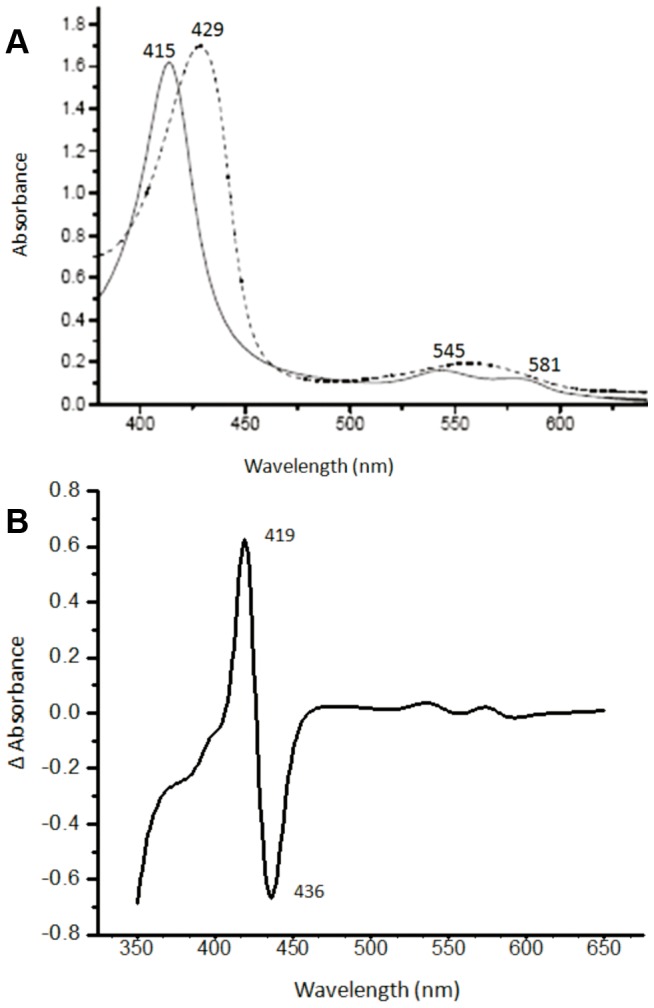
Spectral properties of PheE15Ala mutant of HbN. (A) Optical absorption spectra of oxygenated (solid line) and sodium dithionite reduced species of mutant HbN, recorded in 50 mM Tris.Cl (pH 7.5). (B) CO-difference spectrum of PheE15Ala mutant of HbN. Spectral profile of other PheE15 gate mutants (PheE15Tyr, PheE15Trp and PheE15Trp) appeared similar and matched with the wild type spectrum reported earlier [1].

### Site-directed Mutagenesis and Construction of PheE15 Gate Mutants of HbN

Recombinant plasmid pPRN [3] was used as a source of HbN gene for the site directed mutagenesis. PheE15 mutants to Ala, Tyr, Ile or Trp were generated using a PCR approach. PCR amplified genes were cloned at NdeI-BamHI site of pET28c and expressed under T7 promoter as described previously [3]. Recombinant HbN and its mutant proteins were purified from the cell lysate of *E. coli* using metal affinity chromatography following standard procedures. Authenticity of mutants was confirmed after nucleotide sequencing.

**Table 1:** Oxygen binding and CO association kinetics of PheE15 gate mutants of HbN of *M. tuberculosis.*

Protein	*p* ^50^(O_2_)^a^	*k_on_* (CO)^b^
Wild type	0.019	2.5×10^7^
PheE15Ala	0.021	3.0×10^7^
PheE15Ile	0.016	2.3×10^7^
PheE15Tyr	0.013	2.0×10^7^
PheE15Trp	0.023	1.8×10^7^

a In units of mm Hg.

b M^−1^s^−1^. Values derived from three independent measurements, each consisting of multiple shots (>50) and averaged out by the program to give the final value. The standard deviation is in the range 0.3–0.5 (×10^7^).

**Table 2:** NO-dioxygenase activity of PheE15 gate mutants of HbN.

Protein	NOD activity^a^	% NOD activity
Wild type	27.8±1.3	^100^
PheE15Ala	15.9±1.4	51.1
PheE15Ile	12.6±0.6	45.3
PheE15Tyr	9.9±0.6	34.5
PheE15Trp	8.6±0.4	30.2

The NOD activity of HbN mutants were determined at fixed concentration of NO (1.8 micromole).

a The activity is expressed as nmole NO/heme/s^−1^.

### Measurements of Heme Content and NOD Activity

Total heme content was determined following the procedure described earlier [14]. Heme concentration was calculated from the absorption difference at 556 and 539 nm for the sodium dithionite-reduced and ferricyanide-oxidized sample. NOD activity of cells or purified protein was monitored polarographically as described previously [3], [15]. NO consumption buffer assay contained 60 mM K_2_HPO_4_, 33 mM KH_2_PO_4_, 7.6 mM (NH_4_)_2_SO_4_, 1.7 mM sodium citrate, 10 mM glucose and 200 µg/ml chloramphenicol. NO uptake rate of O_2_-bound HbN and its mutants was measured from the slope of curving traces recorded in the presence of specified concentration of NO following established protocols [3], [15].

**Figure 3:**
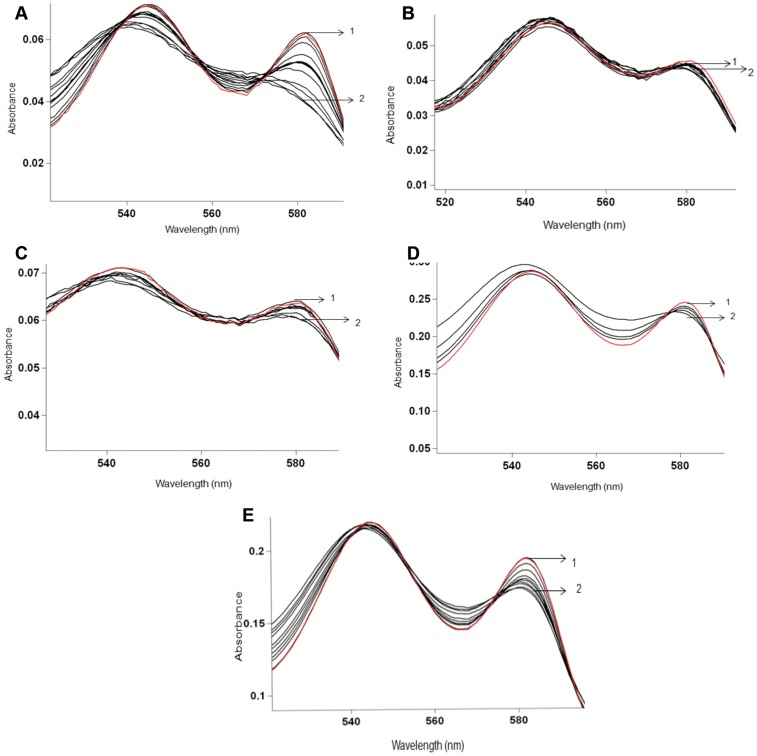
NO oxidation profile of PheE15 gate mutants of HbN. Titration of oxygenated HbN protein (20 µM) was done by adding 15 µM NO sequentially and recording spectra after each addition. Wild type HbN displayed fully oxidized spectra after 12 additions (A), whereas mutants PheE15Tyr (B), PheE15Ile (C), PheE15Trp (D) and PheE15Ala (E) displayed very slow oxidation of the protein and could not be fully oxidized even after 20 additions of NO. The first and last additions are labeled as 1 and 2, respectively.

### Measurement of Ligand Binding

O_2_ and CO binding was checked from the absorption spectra of O_2_- and CO-bound species [9]. CO difference spectra were recorded between 350 to 600 nm after bubbling CO into the protein sample cuvette and recording the difference spectra against sodium-dithionite reduced protein. Oxygen equilibrium curves of HbN mutants were checked following the published procedure [16] to check their *p^50^* value. The association rate for CO binding to HbN mutants was determined by flash photolysis. A concentrated stock solution of deoxyHbN was diluted anaerobically (∼100 µM) into a cuvette (1 mm path length) containing CO (1 mM). The fully liganded sample of ferrous HbN was photodissociated by 0.3 µs excitation pulse from a dye laser. The bimolecular rebinding time courses were collected as described elsewhere [17]. A minimum of five traces were collected and averaged for each experiment.

**Figure 4:**
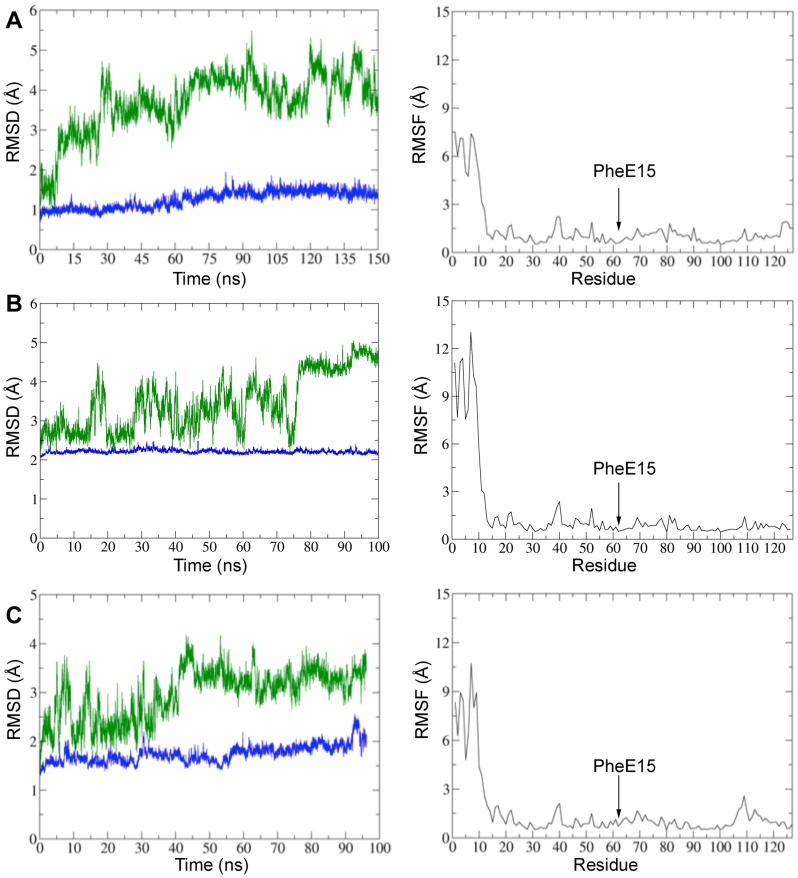
Representation of rmsd and rmsf profiles for PheE15 gate mutants of HbN. (Left) Rmsd (Å) of the protein backbone determined using the X-ray structure (1IDR; subunit A) as reference. The rmsd of the whole protein is shown in green, whereas the rmsd of the residues in the protein core (excluding those in the pre-A segment; residues 1–15) is shown in blue. (Right) Representation of the rmsf (Å) of residues side chains in the protein. The plots correspond to the mutants (A) PheE15Ala, (B) PheE15Ile and (C) PheE15Tyr. The location of the mutated residue Phe(62)E15 is indicated in the plots by an arrow (helix E encompass residues 51–66).

### NO-oxidation by an Oxygenated Adduct of HbN and its Mutants

Wild type and mutant proteins were fully oxygenated by exposing the deoxygenated protein samples to air and checking their absorption spectra, which gave a specific Soret peak at 415 and two peaks, α and β, at 570 and 540 nm, very similar to oxy form of hemoglobin. With a gas tight Hamilton syringe, NO (5 µM) was sequentially added to the oxygenated protein (40 µM), and absorption spectra were recorded after each addition to follow the conversion into the oxidized form. The NO-induced oxidation of mutants was compared with the profile determined for the wild type protein.

**Table 3:** Global similarity index determined by comparison of the motions of the protein backbone in the oxygenated form of wild type HbN and the PheE15 mutants.

ξ_AB_	Wild type	PheE15Ala	PheE15Ile	PheE15Tyr
Wild type	0.68	0.65	0.60	0.62
PheE15Ala		0.67	0.63	0.60
PheE15Ile			0.74	0.64
PheE15Tyr				0.78

The comparison is made considering the 10 most relevant essential motions, which encompass 60–75% of the structural variance along the trajectory.

**Figure 5:**
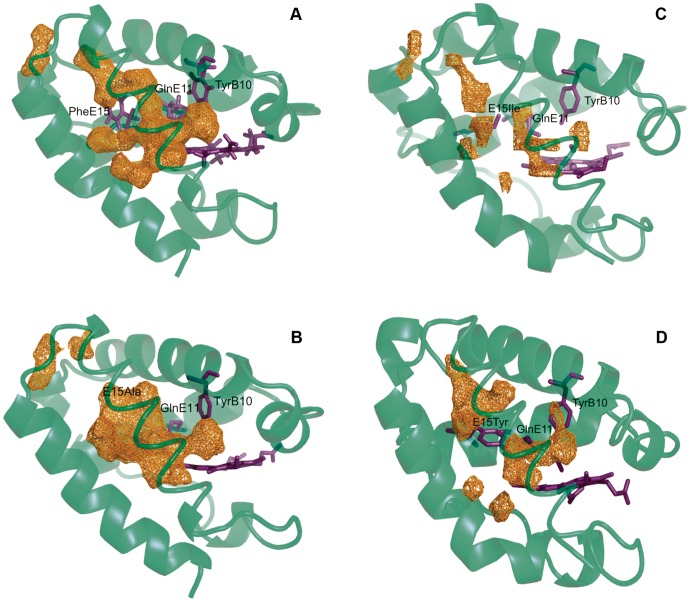
Representation of the accesible volume in wt HbN and its mutants. The accessible volume determined from MDpocket analysis is achieved for a density isocontour of 6.7 in the case of wt protein (A). The use of the same isocontour shows discontinuities in the accessible volume of the tunnel long branch for the different mutants. The disruption is located around the position of the gate in the case of PheE15Ile (C) and PheE15Tyr (D) mutants. For the PheE15Ala species (B) the major disruption involves the region close to the channel entry. Continuous progression of the accessible volume is achieved when the isocontour value is reduced to 5.4 for PheE15Ile and PheE15Tyr, and to 3.4 for PheE15Ala. In the plots the protein backbone corresponds to the energy-minimized structure obtained by averaging the snapshots sampled in the last 0.1 ns for each trajectory.

### Molecular dynamics simulations

The dynamical behaviour of the oxygenated form of HbN mutants was examined by means of extended MD simulations and compared to the results reported in previous studies for the wild type protein [7], [8]. The X-ray structure of wild type *Mtb* HbN (PDB entry 1IDR, chain A, solved at 1.9 Å resolution) was used as starting point for simulations. Mutants were generated by replacing PheE15 by Ala, Ile and Tyr in the X-ray structure of the wild type protein. In all cases simulations were performed using the same protocol adopted in our previous studies [7], [8]. Briefly, the enzyme was immersed in a pre-equilibrated octahedral box of TIP3P [18] water molecules. The final systems contained the protein and around 8600 water molecules (*ca.* 28,270 atoms). The system was simulated in the NPT (1 atm.; 298 K) ensemble using SHAKE [19] to keep bonds involving hydrogen atoms at their equilibrium length, periodic boundary conditions, Ewald sums for treating long range electrostatic interactions [20], and a 1 fs time step for the integration of Newton’s equations. All simulations were performed with the parmm99SB force field [21] and employing heme parameters developed in previous works [7], [8].

**Figure 6:**
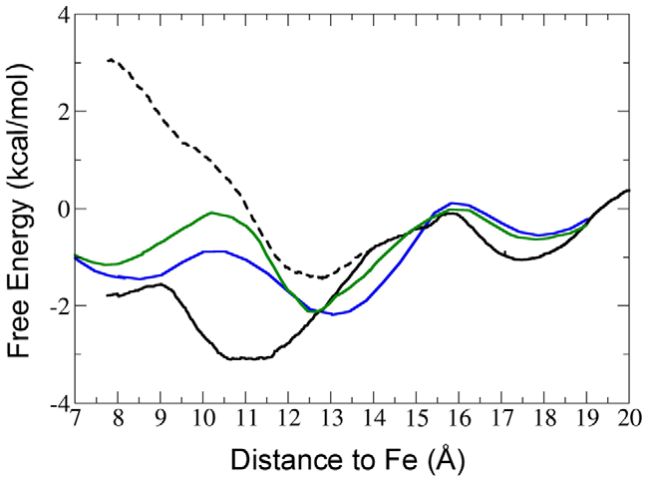
Free energy profile determined for NO migration along the long tunnel branch. MSMD calculations were performed to determine the energetics of ligand migration through the tunnel long branch for Phe15Ile (green) and PheE15Tyr (blue). The profiles are compared with those determined for ligand migration in both *open* (solid line) and *closed* (dashed line) states of oxygenated wt HbN. The free energy is given in kcal/mol, and the distance of the ligand from the heme iron is given in angstroms. For the sake of clarity, error bars are not displayed.

MD simulations were performed with the PMEMD module of the AMBER10 program [22]. The geometry of the models was relaxed by energy minimization carried out in three steps where hydrogen atoms, water molecules and finally the whole system were minimized. Equilibration was performed in successive 50 ps runs where the temperature was gradually increased from 100 K to 298 K in four steps at constant volume, followed by an additional step run at constant pressure for 100 ps. Then, a series of 100–150 ns MD simulations (at 298 K and 1 atm) were run. The analysis of the trajectories was performed using frames collected every 1 ps during the production runs. Furthermore, 75 MD simulations (25 per mutant) were run to explore the pathways for ligand access (free NO in solution) to the heme cavity in PheE15Tyr, PheE15 Ile and PheE15Ala. To this end, five structures of the protein were taken from the last 25 ns of the trajectories run for the oxygenated mutants. These snapshots were used as starting points for unrestrained simulations run in presence of NO, which was placed at random positions around the protein (5 distinct random positions per protein snapshot). The systems were thermalized following the protocol mentioned above, and MD simulations were run up to 20 ns using the same simulation conditions.

**Figure 7:**
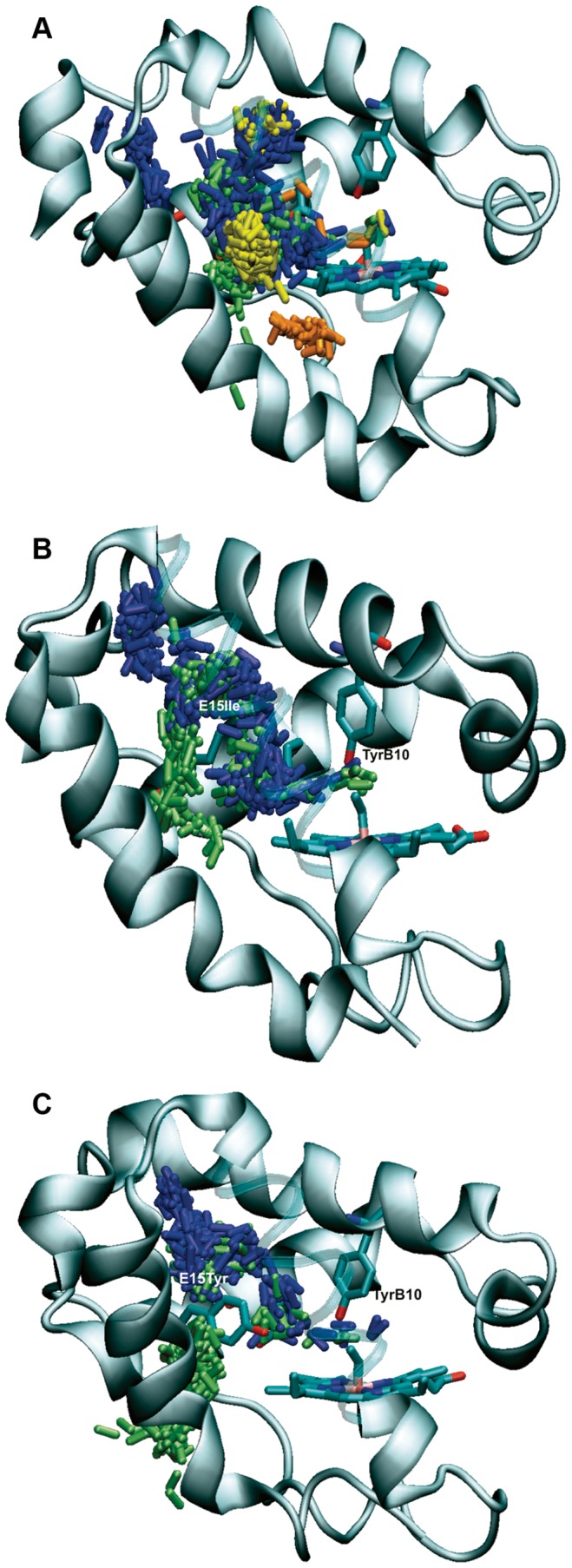
Representative trajectories followed by free NO ligand through the protein matrix. MD simulation of free NO located at random positions from the protein were followed to investigate the pathways leading to the heme cavity for oxygenated forms of (A) PheE15Ala, (B) PheE15Ie and (C) PheE15Tyr. The distinct pathways are indicating by showing the position of NO (represented as sticks) along the trajectory using different colors: long branch (blue), short branch (brown), EH (green), and other (yellow). Note that helix G is displayed as highly transparent cartoon for the sake of clarity.

**Table 4:** Analysis of the migration pathways followed by a free NO in a simulation box containing the solvated proteins PheE15Ala, PheE15Ile and PheE15Tyr.

Mutant	Effective path^a^	Long branch^b^	Shortbranch^c^	EH^d^	Other
PheE15Ala	18 (72.4%)	5	3	7	3
PheE15Ile	21 (85.7%)	15	0	6	0
PheE15Tyr	17 (70.0%)	12	0	5	0

25 independent MD simulations were examined for each mutant in order to determine the migration route followed by NO to reach the heme cavity.

a Fraction of trajectories where the ligand was able to reach the heme cavity in the simulation time. The distinct pathways are displayed in Figure 7 using different colors: long branch (blue), short branch (brown), EH (green), and other (yellow).

b Defined primarily by helices B and E.

c Defined primarily by helices G and H.

d This pathway is defined by residues located in helices E and H.

### Essential Dynamics

The dynamical behavior of HbN and its mutants was explored by means of essential dynamics [23], [24]. Residues 1–15 were excluded as this region is very flexible and would mask the essential motions of the protein core. The backbone atoms were used to superpose the structures sampled in order to derive the essential motions. To this end, for each trajectory the positional covariance matrix of the backbone atoms was built up and diagonalized. The eigenvectors define the type of essential motions of the backbone, and the eigenvalues determine how much of the positional variance in the trajectory is explained by each eigenvector.

### Ligand Migration Profiles

The effect of PheE15 mutation on the migration of ligands was examined using two techniques. The preferred docking sites and migration pathways were identified using MDpocket [25]. Then, the migration free energy profiles were obtained using Multiple Steered Molecular Dynamics (MSMD) [26].

MDpocket is a pocket detection program that uses a fast geometrical algorithm based on a Voronoi tessellation centered on the atoms and the associated alpha spheres, which are clustered and filtered giving origin to pockets and channels [27]. These pockets are then used to identify docking sites and migration paths along the protein matrix. Analyses were performed using 10000 snapshots taken equally spread over the last 50 ns of the trajectories. The minimum and maximum alpha sphere radius was 2.8 Å and 5.5 Å, respectively. The identified cavities were superposed in time and space and a density map was generated from this superposition. Stable cavities are identified as high-density 3D isocontours, while low-density isocontours denote transient or nearly non-existent cavities in the MD simulation.

MSMD simulations were run to evaluate the free energy profiles of ligand migration through the tunnels using Jarzynski’s equality [26], which estimates the free energy from an ensemble of irreversible works along the same reaction coordinate. A steering potential forces the motion of the probe with constant velocity along the reaction coordinate. The reaction coordinate was the iron-ligand distance, the force constant was 200 kcal mol^−1^ Å^−1^ and the pulling velocity 0.025 Å ps^−1^. The free energy profile of ligand migration along a tunnel was obtained following the computational scheme reported in previous studies [8]. At least twenty SMD simulations were run pushing the ligand through the tunnel from the solvent towards the iron. The starting snapshot for each SMD was taken from the final structure of an equilibrated MD simulation with the ligand placed at a fixed distance from the iron. Typically those distances are chosen from the preferred docking sites found in MDpocket analysis in conjunction with short MD simulations run to examine the motion of the free diatomic ligand along the tunnel.

## Results and Discussion

Four gate mutants, where PheE15 in wild type (wt) HbN was replaced by Ala (PheE15Ala), Ile (PheE15Ile), Tyr (PheE15Tyr) and Trp (PheE15Trp), were created by site-directed mutagenesis. Mutations were chosen to span a wide range of sizes, varying from the small methyl group in Ala to the large indole ring in Trp, the replacement of the planar benzene by the branched chain of Ile, and the conservative mutation of PheE15 by Tyr. It was expected that the distinct chemical nature of the side chains would translate into differences in the ligand migration through the long tunnel, which in turn should lead to differences in the NOD activity measured for the mutants. Thus, in the absence of relevant structural alterations in the tunnel due to the mutations at position E15, which might be relevant for the bulky Trp, it was expected that replacement of PheE15 to Ala should open permanently the long tunnel, whereas mutation to Trp should occlude the access of ligands. Likewise, the branched side chain of Ile was expected to limit the accessibility of diatomic ligands through the tunnel. Finally, the conservative PheE15Tyr mutation was *a priori* expected to have little effect on the migration properties.

### Effect of Mutations at PheE15 Gate of HbN on O_2_/CO Binding

Absorption spectra of O_2_ and CO bound forms of mutants were indistinguishable from that of wt HbN (Fig. 2), suggesting that all these mutants bind O_2_. The ability to bind O_2_ was further assessed by measuring the *p* ^50^ values (Table 1). For the wt protein, the *p* ^50^ value was 0.019 (mm Hg), which compares well with previous data [1]. When the PheE15 gate mutants were compared with wt HbN, no significant difference in their *p* ^50^ profile was observed suggesting that the O_2_ binding properties of mutants are not impaired. To substantiate these results, the rate of CO association was also determined to characterize the kinetics of ligand association (Table 1). The *k_on_* value determined for the wt protein (2.5×10^7^ M^−1^ s^−1^) is slightly larger than the value reported by Couture *et al.* (0.657×10^7^ M^−1^ s^−1^) [1]. Nevertheless, all the mutants displayed CO association rates comparable to that of wt HbN, indicating that mutation at PheE15 residue does not affect the O_2_/CO binding properties of HbN.

### Mutations at PheE15 Gate of HbN Alter its NOD Activity

Even though mutants exhibit very similar O_2_ binding properties, relevant differences were observed in their NO metabolizing activities (Table 2). The NOD activity of PheE15Ile was reduced to around 55% of the activity measured for the wt enzyme, whereas a larger reduction (around 70%) in NOD activity was observed for the PheE15Trp mutant. Unexpectedly, mutation of PheE15 to Tyr also yielded a significant reduction (around 65%) in the NOD activity, suggesting that the apparently conservative replacement of benzene by phenol has a drastic influence on the ligand migration properties of the mutant. Finally, the PheE15Ala mutant also exhibited a significant reduction (around 49%) in the NOD activity.

### Oxidation of NO by Oxygen Adduct of PheE15 Gate Mutants of HbN

The NO oxidation profile of wt HbN and its mutants was determined by titrating the oxy forms of protein with NO in a time course manner (Fig. 3). The addition of NO (5 µm) resulted in the appearance of a partially oxidized spectrum of HbN and repeated addition of NO solution to this sample resulted in a fully oxidized spectrum shifting Soret peak of oxyHbN from 415 to 405 nm. In contrast, similar additions of NO to the oxygenated form of mutants PheE15Trp, PheE15Ile and PheE15Tyr did not change the spectra to the oxidized form immediately and changed slowly after 30–35 min of exposure, indicating very slow NO oxidation. Finally, NO oxidation by PheE15Ala mutant displayed a spectral profile similar to PheE15Ile and PheE15Tyr, which is intermediate between those observed for the wt protein and the PheE15Trp mutant.

### MD Simulations

The preceding data indicate that the different PheE15 mutations do not affect the binding of O_2_ to the heme. However, since the mutants exhibit a distinctive reduction in the NOD activity, the PheE15 residue has to play a key role in mediating the access of NO to the oxygenated protein. On the basis of these findings, MD simulations were run with a twofold purpose: to examine the structural integrity of the overall protein fold, and to identify local changes in the long branch tunnel that might affect the ligand migration. To this end, a series of 100–150 ns MD simulations were run for the heme-bound O_2_ forms of PheE15Ala, PheE15Ile and PheE15Tyr mutants, and the results were compared with those obtained for the wt protein. This simulation time has been shown suitable to describe the conformational transitions of PheE15 in our previous studies for the wt HbN [7]–[9]. Choice of the simulation time, however, was also dictated by consistency in the analysis of the structural and dynamical properties of the simulated systems, either related to the overall features of the protein backbone or to the ligand migration through the protein matrix. Such a convengence was achieved for all the simulated systems but for the PheE15Trp mutant (see below).

For the particular case of the PheE15Tyr mutant, two MD simulations differing in the starting orientation of TyrE15 were run. Thus, on the basis of the conformational preferences found for PheE15 in both the X-ray structure [5] and previous MD studies [7], [8], the phenol ring was oriented in the closed or open conformations, which prevent or facilitate ligand migration through the tunnel, respectively. When TyrE15 was in the closed orientation, the side chain remained stable, and no transitions between open and closed states were found along the whole trajectory (Fig. S1). There was only a conformational change leading to the transient formation of a hydrogen bond between the hydroxyl group of TyrE15 and the carbonyl group of Ile115. When the MD simulation started from the open conformation, the side chain of TyrE15 suddenly changed to the closed conformation after the first 12 ns and then remained stable until the end of the trajectory (Fig. S1), even though few attempts to form transient hydrogen-bond interactions with Ile115 can be observed. Since the two simulations exhibited the same behavior, hereafter discussion of PheE15Tyr mutant will be limited to the trajectory starting from the closed conformation.

Finally, the MD simulation run for the PheE15→Trp mutant was extended up to 200 ns. However, the results will not be presented here as the analysis of the trajectory points out that the structural changes induced by the mutation both in the tunnel and in the global protein structure are still not fully converged. It seems that a proper description of the structural rearrangements triggered by this mutation would require longer simulations, making it necessary to be cautious for not overinterpreting the structural changes due to the PheE15→Trp mutation, which cannot be easily accommodated in the tunnel (see Fig. S2 for details).

### Structural Analysis

For all the simulations, inspection of both the time evolution of the potential energy (Fig. S3) and the rmsd (Fig. 4; see also Fig. S4) determined for the backbone atoms of the protein core (excluding the first 15 residues) supports the integrity of the simulated proteins. Thus, the rmsd of the protein core (ranging from 1.2 to 2.1 Å for the different mutants) remains very stable after the first few nanoseconds. In contrast, a much larger rmsd profile showing notable fluctuations along the trajectory is obtained when the whole protein backbone is included in the analysis. This finding indicates that the pre-A segment is very flexible in all the mutants and can adopt a large number of conformational states, as noted in the formation of distinct structural arrangements for residues 1–15 (data not shown). Therefore, the mutation does not alter the large conformational flexibility found for the pre-A segment in the wt HbN [9], [28].

The geometrical arrangement of residues TyrB10 and GlnE11 in the distal cavity is a structural feature of particular relevance, as it has been proposed that opening of the gate is modulated by the oxygen-sensing properties of the TyrB10-GlnE11 pair [8], [29]. In the oxygenated wt HbN these residues form a network of hydrogen bonds, where the hydroxyl group of TyrB10 is hydrogen-bonded to the heme-bound O_2_, and also accepts a hydrogen bond from the side chain of GlnE11. Due to these interactions, the GlnE11 side chain lies closer to the benzene ring of PheE15 than in the deoxygenated protein, and the enhanced steric clash favors opening of the PheE15 gate upon O_2_ binding. Accordingly, it might be argued that disruption of the hydrogen bonds formed by TyrB10 and GlnE11 could explain the reduction in NOD activity of the mutants. However, the analysis of the trajectories completely rules out this possibility (Fig. S5). Thus, in all the mutants the hydroxyl group of TyrB10 is hydrogen-bonded to the heme-bound O_2_ (average distances about 2.85 Å), and the side chain amide nitrogen of GlnE11 is hydrogen-bonded to the TyrB10 hydroxyl group (average distances about 3.0 Å). These interactions reproduce the hydrogen bonds found in the X-ray structure, as the corresponding distances (averaged for subunits A and B) are 3.15 and 2.95 Å. Therefore, the hydrogen-bond network found in wt HbN is not affected by the PheE15 mutations, and hence one should expect that the steric pressure exerted by GlnE11 is retained in the mutated proteins.

### Dynamical Analysis of the Protein Backbone

Binding of O_2_ to the heme also changes the dynamical motion of the protein backbone [7], [8]. Thus, essential dynamics analysis of oxygenated HbN reveals that the major motions in the deoxygenated protein affect helices C, G and H, while the largest contribution to protein flexibility comes from helices B and E in the oxygenated protein. Since helices B and E define the walls of the tunnel long branch, the increased motion of these helices should facilitate the transition between open and closed states of the gate, thus influencing the ligand migration through the tunnel. This dynamical alteration agrees with the large scale conformational change observed experimentally upon binding of NO to the ferric form of wt HbN [30], and with the occurrence of distinct conformational relaxation processes found in the kinetics of CO recombination to the protein encapsulated in gels [31].

In order to investigate whether mutation of the PheE15 gate influences the dynamics of the protein backbone and eventually affects the migration of NO in the oxygenated protein, we determined the essential dynamics of the mutants and compared them with the wt protein. Diagonalization of the positional covariance matrix for the backbone atoms points out that few motions account for a significant fraction of the protein dynamics. Nearly 50% and 70% of the backbone conformational flexibility is accounted for by the first 4 and 10 principal components (Table S1). The two first essential motions, which mainly involves motions of helices B, E and H, loop F and the hinge region around helix C, account for 22–37% of the structural variance in the backbone of the mutants, in agreement with the value (36%) determined for wt HbN.

The similarity between the structural fluctuations of the protein backbone in wt HbN and its mutants was measured by means of the similarity index 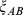 (see Text S1), which takes into account the nature of the essential motions and their contribution to the structural variance of the protein [32]. When the 10 most relevant motions are considered, the similarity index varies in the range 0.60–0.65, which is slightly lower than the self-similarities obtained for wt HbN and mutants (Table 3). These results point out that mutations preserve to at large extent the dynamical behavior of the protein. For the sake of comparison, the similarity indexes determined for the 10 most essential motions between wt HbN and the mutants TyrB10Phe and GlnE11Ala only amount to 0.47 and 0.38, respectively [27], indicating that the hydrogen-bond network formed by the TyrB10-GlnE11 pair has a larger impact on the protein dynamics than mutation of PheE15.

### Ligand Migration

Since the preceding results did not reveal significant changes neither in the hydrogen-bond network of TyrB10-GlnE11 pair nor in the dynamical behavior of the protein skeleton, the reduction in NOD activity determined for PheE15 mutants might reflect a reduced accessibility of ligands for migrating through the long tunnel. To corroborate this hypothesis, we determined the shape of internal tunnels by using MDpocket, which provides a grid that encloses the protein, where each grid point is assigned an occupancy value that denotes the accessibility of the volume associated to that point [23].

For the wt HbN the tunnel long branch appears as a continuous cavity leading from the protein surface to the heme cavity, as noted by the continuous progression of the accessible volume isocontour shown in Fig. 5. In contrast, the representation of the same isocontour for the mutants reveals a discontinuous channel, even in the case of the mutation to Ala.

For mutants PheE15Ile and PheE15Tyr, such a disruption affects the region that surrounds the mutated residue, suggesting that ligand migration through the tunnel long branch is impeded by the steric hindrance imposed by the side chains of the mutated residues (Fig. 5C,D). This is confirmed by the free energy profiles determined by MSMD calculations for these mutants, where the limiting step for ligand migration is associated to surpassing the gate (Fig. 6). In fact, the free energy profiles are intermediate between those obtained for the wt HbN with the PheE15 gate in either closed or open conformational states. Compared to the wt protein in the open state, mutation of the gate slightly destabilizes the minimum located at around 11 Å (i.e., the docking site before the gate), and increases the barrier required to pass above the side chain of the mutated residue in order to access the heme cavity. Therefore, a small diatomic ligand such as NO is expected to be trapped effectively at the highly hydrophobic entrance of the long branch, thus enhancing the local concentration, but access to the heme cavity is mainly limited by the hindrance due to the side chain of the mutated gate.

In contrast with the preceding findings, the isocontour is continuous around the mutated gate in the PheE15Ala mutant, as expected from the small side chain of Ala. However, compared to the wt protein (Fig. 5A), there is a significant occlusion at the entrance of the channel (Fig. 5B), which stems from a slight readjustment of helices B and G that reduces the width of the channel entry by 0.4–0.6 Å (as measured from distances between Cα atoms of residues located at the helical ends). Accordingly, the probability of the ligand to be trapped at the entrance of the tunnel long branch is lower compared to wt HbN. In turn, this finding raises the question about the existence of alternative pathways that can justify the remaining NOD activity (around 51% compared to wt protein) retained by PheE15Ala mutant. To this end, we examined the trajectory followed by a free NO in a water box containing the PheE15Ala mutant and determined the routes leading to the heme cavity in 25 independent MD simulations. The results (see Table 4) showed that 18 out of 25 trajectories were successful in allowing the ligand (NO) to achieve the heme cavity within the simulation time. Nevertheless, only 5 trajectories showed that NO migrates via the tunnel long branch, and in 7 simulations the entry pathway involved the EH tunnel (defined by residues in helices E and H) reported by Daigle et al. [33]. Remarkably, in 3 trajectories NO was able to reach the heme cavity through the short branch, and in other 3 trajectories NO accessed the heme cavity via a distinct channel (see Fig. 7A). Thus, even though the reduction in NOD activity can be related to the occlusion of ligand access at the beginning of the tunnel long branch, this effect is counterbalanced by the existence of three alternative entry pathways, which arise from slight structural alterations in the protein skeleton. This finding reveals the delicate structural balance imposed by the PheE15 gate, which not only regulates ligand migration, but also contributes to avoid the collapse of helices B and E, thus preserving the structural integrity and ligand accessibility along the tunnel long branch.

For the sake of completeness, this analysis was also performed for PheE15Ile and PheE15Tyr mutants. For these mutants the access routes leading to the heme cavity were significantly different. Thus, the trajectories primarily involved migration through the tunnel long branch (Table 4), which should be considered the main pathway for ligand migration in the oxygenated protein. In this pathway, the ligand remained docked in a region before the gate for significant periods of time (as noted in the high density of NO molecules located before the mutated gate residue; see Fig. 7B,C) until it was able to surpass the barrier due to the side chain of Ile/Tyr and access the heme cavity. There were some attemtps to get the heme cavity through the short tunnel, but they were unsuccessful. Finally, only in very few cases the ligand entered the protein through the tunnel EH, leading to the docking site located before the mutated gate residue. Therefore, these findings are in contrast with previous modeling studies where the short tunnel was reported to be the main route for NO diffusion [34]. Furthermore, present results indicate that the main route in PheE15Ile and PheE15Tyr is the tunnel long branch, which supports the role of the tunnel long branch in the dual-path migration mechanism [7], [8] and particularly the functional relevance of the PheE15 gate.

### Conclusion

The experimental data collected for the mutants indicate that the PheE15 mutation does not affect the binding of O_2_ to the heme, as noted in the similar absorption spectra of O_2_ and CO bound forms, as well as in the similar *p* ^50^ and *k_on_* values determined for both wt and mutated proteins. These findings suggest that the PheE15 mutation has little impact on the tunnel short branch, which is proposed to be the main pathway for migration of O_2_ to the heme cavity. However, since the mutants exhibit a distinctive reduction in the NOD activity, PheE15 residue has to play a key role in mediating the access of NO to the oxygenated protein. In agreement with the dual-path migration mechanism [7], [8], the main pathway for NO migration is the tunnel long branch. This finding points out the delicate structural balance imposed by the PheE15 gate, which not only regulates ligand migration, but also contributes to avoid the collapse of helices B and E, thus preserving the ligand accessibility along the tunnel long branch. Overall, the results presented herein demonstrate the pivotal role of PheE15 in modulating the NOD function of Mtb HbN, thus confirming the suggestion by Milani and coworkers [5] about the gating role of PheE15 in HbN.

## Supporting Information

Figure S1 **Representation of the conformational orientation of the TyrE15 side chain in the two trajectories run for the PheE15Tyr mutant.** Time (ns) evolution of the dihedral angle H-Cα-Cβ-Cδ (degrees) of TyrE15 in the simulation started by placing the phenol ring in (top) open and (bottom) closed conformations, following the two main orientations found for the side chain of PheE15 gate in wild type HbN [7], [8].
(TIF)

Figure S2 **Representation of structural changes for the PheE15Trp mutant.** (Top) Superposition of the backbone of the snapshots sampled at 40 (green) and 180 (orange) ns along the trajectory run for the PheE15Trp mutant. The plot shows the drastic change in the orientation of the Trp side chain, the displacement of the heme, and the structural rearrangement of several helices. For the sake of clarity, helix G is hown as ribbon. (Bottom) Time (ns) evolution of the dihedral angle (degrees) that determines the orientation of the indole ring of Trp.
(TIF)

Figure S3 **Representation of the potential energy for simulated systems.** Time (ns) evolution of the potential energy (x 10^3^; kcal/mol) for the simulations of the oxygenated PheE15 mutants. Top: (left) PheE15Ala; (right) PheE15Ile. Bottom: PheE15Tyr in the simulation started by placing the Tyr side chain in (left) closed and (right) open conformations.
(TIF)

Figure S4 **Representation of rmsd and rmsf profiles for** **PheE15Tyr gate mutant of HbN (trajectory started from open conformation).** (Left) Rmsd (Å) of the protein backbone determined using the X-ray structure (1IDR; subunit A) as reference. The rmsd of the whole protein is shown in green, and the rmsd of the residues in the protein core (excluding the pre-A segment; residues 1–15) is shown in blue. (Right) Representation of the rmsf (Å) of residues in the protein. The plots correspond to the trajectory run started by placing the side chain of TyrE15 in the open conformation.
(TIF)

Figure S5 **Representation of hydrogen-bond distances for the TyrB10-GlnE11.** Time (ns) evolution of distances (Å) from the TyrB10 hydroxyl oxygen to the heme-bound O_2_ and from the GlnE11 side chain amide nitrogen to the TyrB10 hydroxyl oxygen are shown in blue and green, respectively. Top: (left) PheE15Ala; (right) PheE15Ile. Bottom: PheE15Tyr in the simulation started by placing the Tyr side chain in (left) closed and (right) open conformations.
(TIF)

Table S1 Structural variance (%) of the protein backbone. The contribution of the first essential motions to the structural variance is gindicated for the mutated forms of HbN. The cumulative value is given in parenthesis.
(DOCX)

Text S1 Global similarity between two sets of essential eigenvectors.
(DOCX)
