# Supplementary figures and images for: Role of PheE15 Gate in Ligand Entry and Nitric Oxide Detoxification Function of Mycobacterium tuberculosis Truncated Hemoglobin N
Source: PLoS One. 2012 Nov 8;7(11):e49291. doi: 10.1371/journal.pone.0049291 (PMC3493545; doi:10.1371/journal.pone.0049291)

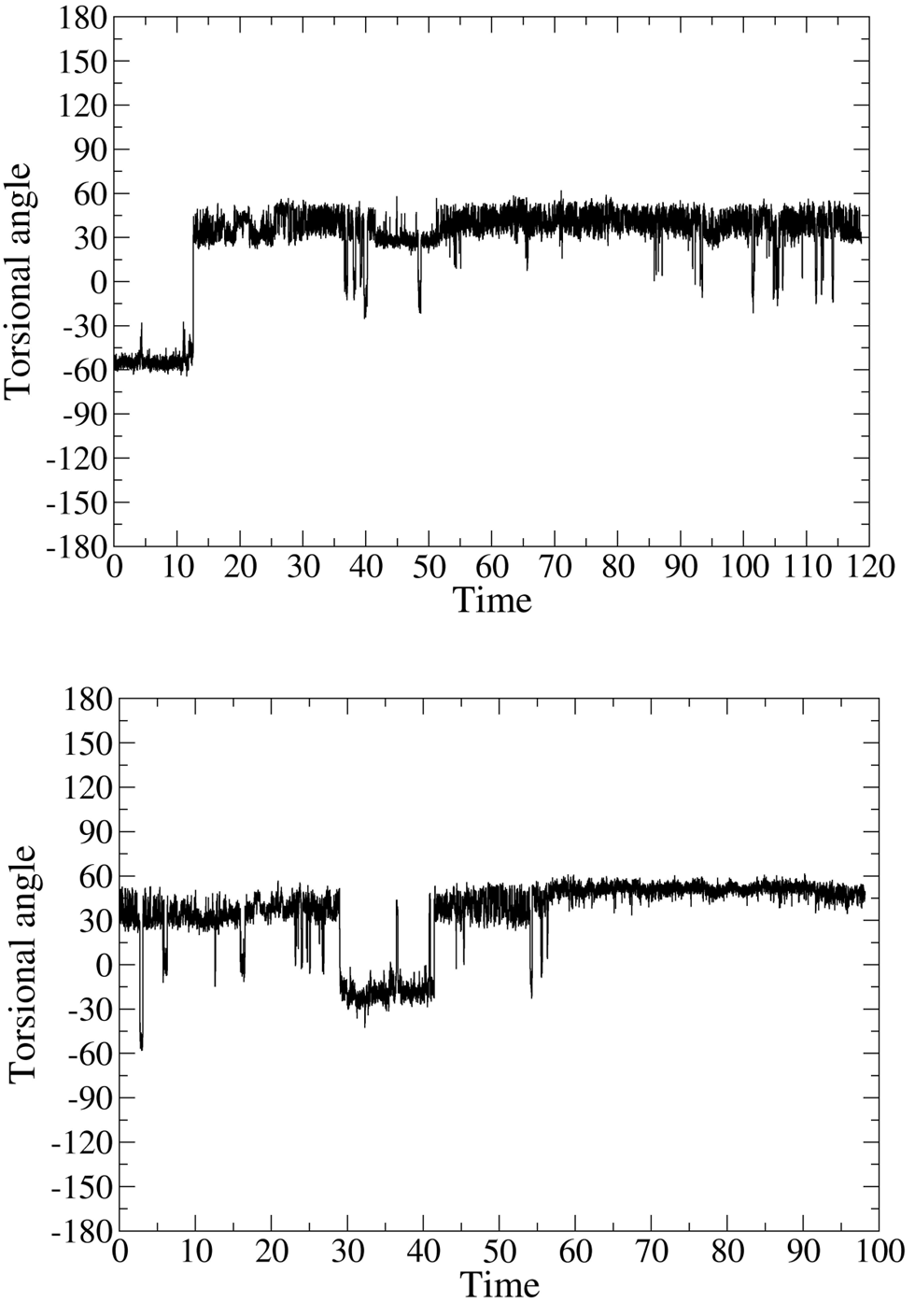

Supplement: Figure S1 — Representation of the conformational orientation of the TyrE15 side chain in the two trajectories run for the PheE15Tyr mutant. Time (ns) evolution of the dihedral angle H-Cα-Cβ-Cδ (degrees) of TyrE15 in the simulation started by placing the phenol ring in (top) open and (bottom) closed conformations, following the two main orientations found for the side chain of PheE15 gate in wild type HbN [7], [8]. (TIF) [file pone.0049291.s001.tif]

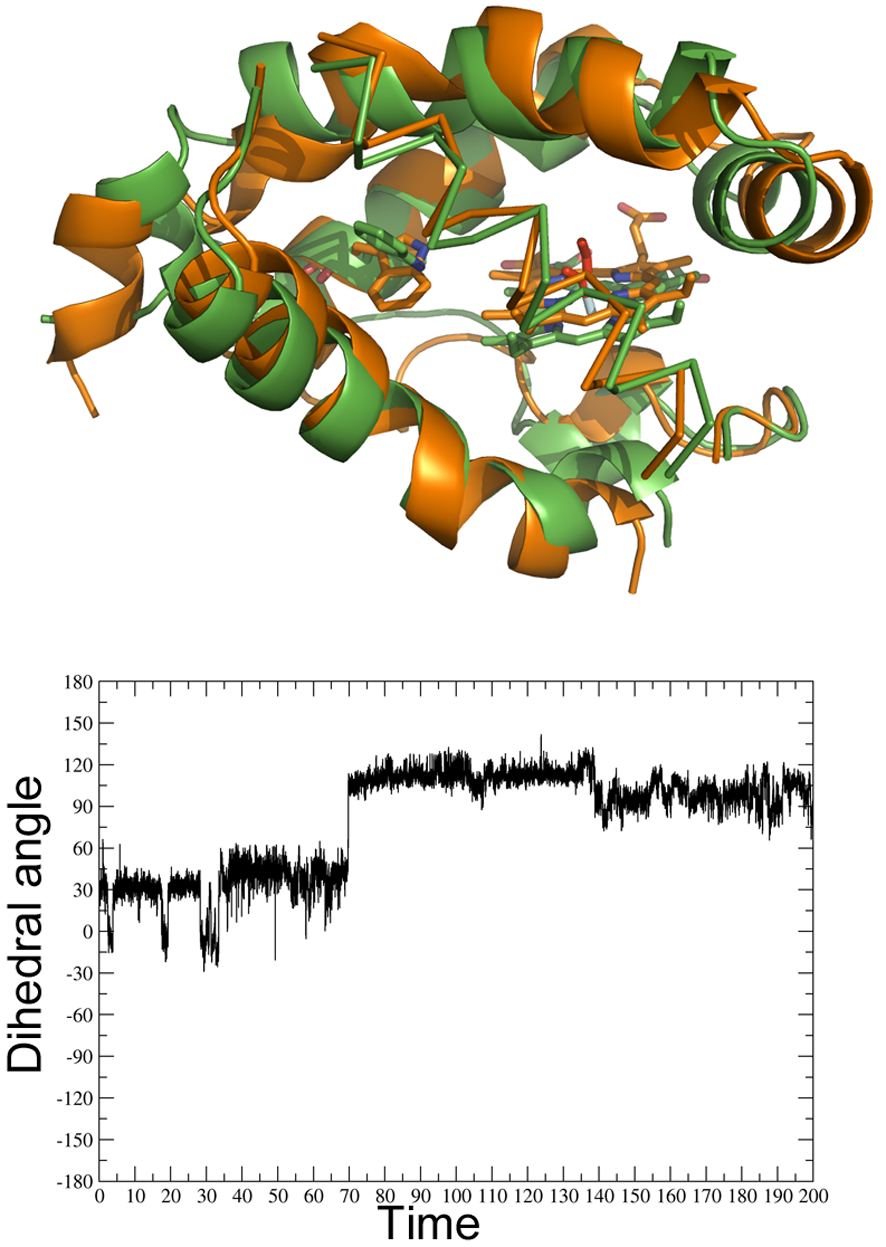

Supplement: Figure S2 — Representation of structural changes for the PheE15Trp mutant. (Top) Superposition of the backbone of the snapshots sampled at 40 (green) and 180 (orange) ns along the trajectory run for the PheE15Trp mutant. The plot shows the drastic change in the orientation of the Trp side chain, the displacement of the heme, and the structural rearrangement of several helices. For the sake of clarity, helix G is hown as ribbon. (Bottom) Time (ns) evolution of the dihedral angle (degrees) that determines the orientation of the indole ring of Trp. (TIF) [file pone.0049291.s002.tif]

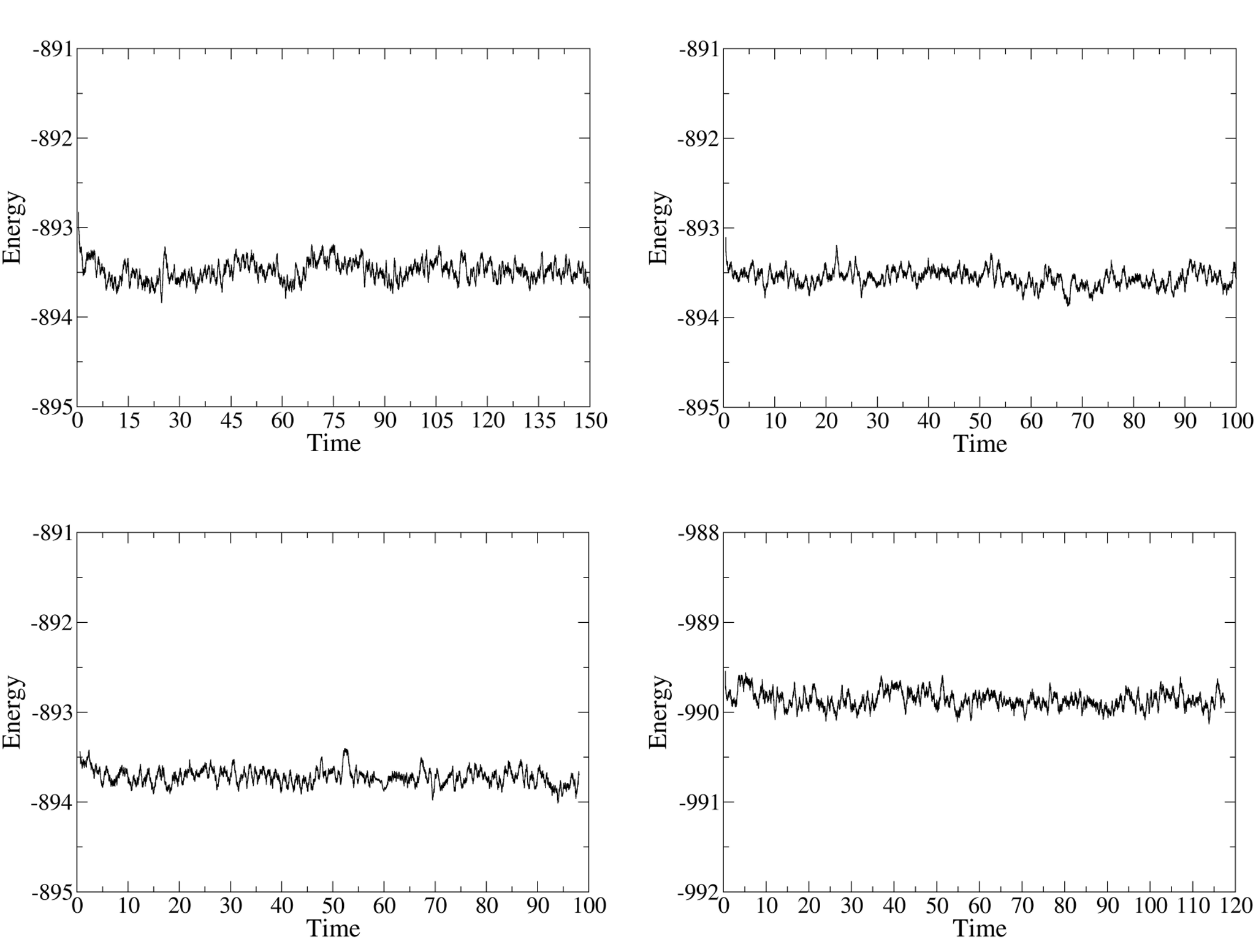

Supplement: Figure S3 — Representation of the potential energy for simulated systems. Time (ns) evolution of the potential energy (x 103; kcal/mol) for the simulations of the oxygenated PheE15 mutants. Top: (left) PheE15Ala; (right) PheE15Ile. Bottom: PheE15Tyr in the simulation started by placing the Tyr side chain in (left) closed and (right) open conformations. (TIF) [file pone.0049291.s003.tif]

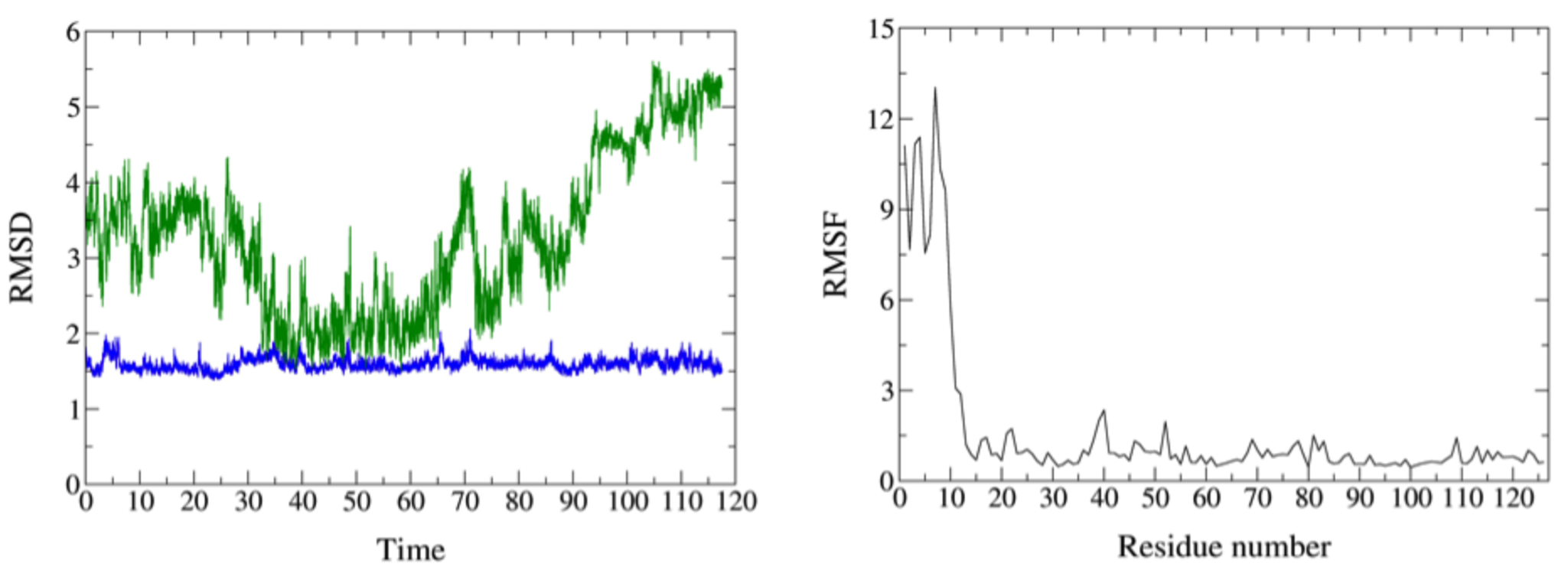

Supplement: Figure S4 — Representation of rmsd and rmsf profiles for PheE15Tyr gate mutant of HbN (trajectory started from open conformation). (Left) Rmsd (Å) of the protein backbone determined using the X-ray structure (1IDR; subunit A) as reference. The rmsd of the whole protein is shown in green, and the rmsd of the residues in the protein core (excluding the pre-A segment; residues 1–15) is shown in blue. (Right) Representation of the rmsf (Å) of residues in the protein. The plots correspond to the trajectory run started by placing the side chain of TyrE15 in the open conformation. (TIF) [file pone.0049291.s004.tif]

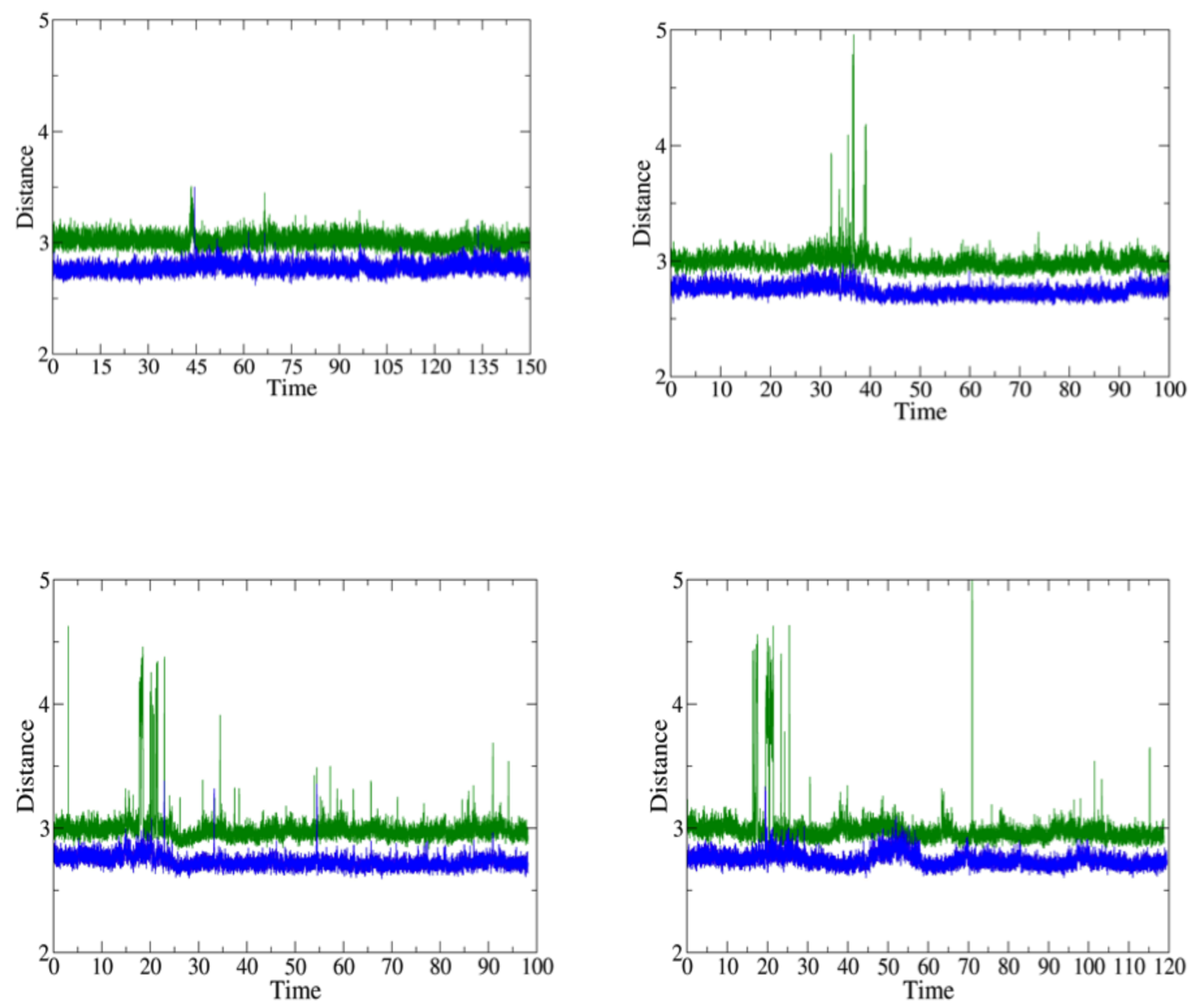

Supplement: Figure S5 — Representation of hydrogen-bond distances for the TyrB10-GlnE11. Time (ns) evolution of distances (Å) from the TyrB10 hydroxyl oxygen to the heme-bound O2 and from the GlnE11 side chain amide nitrogen to the TyrB10 hydroxyl oxygen are shown in blue and green, respectively. Top: (left) PheE15Ala; (right) PheE15Ile. Bottom: PheE15Tyr in the simulation started by placing the Tyr side chain in (left) closed and (right) open conformations. (TIF) [file pone.0049291.s005.tif]
