# Supplementary material for: Role of PheE15 Gate in Ligand Entry and Nitric Oxide Detoxification Function of Mycobacterium tuberculosis Truncated Hemoglobin N
Source: PLoS One. 2012 Nov 8;7(11):e49291. doi: 10.1371/journal.pone.0049291 (PMC3493545; doi:10.1371/journal.pone.0049291)
Supplement: Table S1 — Structural variance (%) of the protein backbone. The contribution of the first essential motions to the structural variance is gindicated for the mutated forms of HbN. The cumulative value is given in parenthesis. (DOCX) [file pone.0049291.s006.docx]

**Table S1**. Structural variance (%) of the protein backbone accounted for by the first essential motions for the mutated HbN. The cumulative value is given in parenthesis.

| **Motion** | PheE15Ile | PheE15Tyr  (closed) | PheE15Tyr  (open) | PheE15Ala |
| --- | --- | --- | --- | --- |
| 1 | 13.1 (13.1) | 24.3 (24.3) | 15.4 (15.4) | 16.3 (16.3) |
| 2 | 10.0 (23.1) | 10.8 (35.1) | 12.1 (27.5) | 15.1 (31.4) |
| 3 | 7.5 (30.6) | 6.6 (41.7) | 7.6 (35.1) | 11.2 (42.6) |
| 4 | 6.9 (37.5) | 5.9 (47.6) | 6.5 (41.6) | 5.9 (48.5) |
| 10 | 3.0 (60.3) | 2.5 (67.2) | 2.6 (67.2) | 2.4 (68.0) |
