# Supplementary material for: Role of PheE15 Gate in Ligand Entry and Nitric Oxide Detoxification Function of Mycobacterium tuberculosis Truncated Hemoglobin N
Source: PLoS One. 2012 Nov 8;7(11):e49291. doi: 10.1371/journal.pone.0049291 (PMC3493545; doi:10.1371/journal.pone.0049291)
Supplement: Text S1 — Global similarity between two sets of essential eigenvectors. (DOCX) [file pone.0049291.s007.docx]

**Similarity index**

The global similarity between two sets of essential eigenvectors is determined using the following equation:


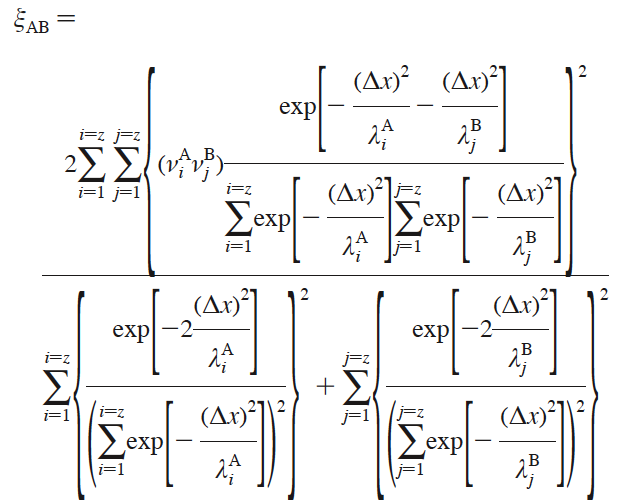


where $\lambda_{i}$ is the eigenvalue (in Å^2^) associated with eigenvector $\mu_{i}$, whose unitary vector is $\nu_{i}$.

Perez, A., Blas, J. R., Rueda, M., Lopez-Bes, J. M., de la Cruz, X. and Orozco, M. (2005) Exploring the essential dynamics of B-DNA. *J. Chem. Theory Comput*. **1**, 790-800.
